# The Effect of Continuous Positive Airway Pressure Therapy on Obstructive Sleep Apnea-Related Hypertension

**DOI:** 10.3390/ijms22052300

**Published:** 2021-02-25

**Authors:** Ronni Baran, Daniela Grimm, Manfred Infanger, Markus Wehland

**Affiliations:** 1 Department of Biomedicine, Aarhus University, 8000 Aarhus, Denmark; 201709730@post.au.dk; 2 Department for Microgravity Research and Translational Regenerative Medicine, Otto-von-Guericke University, D-39106 Magdeburg, Germany; manfred.infanger@med.ovgu.de

**Keywords:** obstructive sleep apnea, hypertension, CPAP

## Abstract

Obstructive sleep apnea (OSA) is a common disease, with approximately 3–7% of men and 2–5% of women worldwide suffering from symptomatic OSA. If OSA is left untreated, hypoxia, microarousals and increased chemoreceptor stimulation can lead to complications like hypertension (HT). Continuous positive airway pressure (CPAP) is the most common treatment for OSA, and it works by generating airway patency, which will counteract the apnea or hypopnea. More than one billion people in the world suffer from HT, and the usual treatment is pharmacological with antihypertensive medication (AHM). The focus of this review will be to investigate whether the CPAP therapy for OSA affects HT.

## 1. Introduction

Obstructive sleep apnea (OSA) is the most prevalent type of sleep apnea, and it is a common disease worldwide as well [1]. The most common treatment for OSA is continuous positive airway pressure (CPAP), and this treatment gives a significantly higher health-related quality of life the more adherent patients are [2]. If OSA is left untreated, it can result in complications like hypertension (HT), which can lead to other and more severe health problems like heart failure, stroke and premature death [3]. HT is defined by the European Society of Hypertension (ESH) as systolic blood pressure (SBP) values > 140 mmHg and/or diastolic blood pressure (DBP) values > 90 mmHg. As estimated in 2015, HT affects around 1.13 billion people worldwide [4,5].

The standard treatment of HT is either lifestyle changes or pharmacological therapy, and especially pharmacological treatment has some contraindications and adverse effects (AEs). Angiotensin receptor blockers (ARBs), for example, may increase the risk of adverse drug reactions [4,6]. As OSA leads to secondary HT, the focus of this review is to investigate if CPAP treatment of OSA affects HT.

## 2. Obstructive Sleep Apnea

OSA is a sleep-related breathing disorder, and the cause of OSA is a repeated obstruction of the upper airways during sleep, which lasts at least 10 s but can continue for more than a minute. During the obstructive respiratory events, oxygen saturation will decrease, but it will usually return to normal values again. Between 3–7% of men and 2–5% of women suffer from OSA-associated daytime sleepiness, while approximately 24% of men and 9% of women suffer from OSA without daytime sleepiness. According to the American Academy of Sleep Medicine (AASM), the diagnostic criteria of OSA are five or more obstructive respiratory events per hour of sleep and at least one of the following symptoms: sleepiness during the day, loud snoring, interruptions of breathing during sleep or the patient has been diagnosed with a mood disorder, cognitive dysfunction, stroke, atrial fibrillation, type II diabetes mellitus or HT, among others. OSA can also be diagnosed if there are >15 obstructive respiratory events per hour of sleep [7].

The degree of OSA can be measured with the Apnea-Hypopnea Index (AHI), which is divided into four degrees of severity based on the number of apneas and hypopneas per hour of sleep as seen in Table 1 [8,9].

One way to assess daytime sleepiness quantitatively is by using the Epworth Sleepiness Scale (ESS), which is a subjective questionnaire about how sleepy a person feels in different situations during the day. The patients rate their sleepiness from 0 (would never doze during this activity) to 3 (high chance of dozing during this activity), for eight different activities like watching TV, sitting and talking with someone or riding as a passenger in a car for an hour without a break [10].

### 2.1. Causes of Obstructive Sleep Apnea

#### 2.1.1. Gender 

As already mentioned, men have a higher prevalence of OSA than women and, therefore, it seems like men are predisposed to OSA. This was shown in Pływaczewski et al., who reported a nearly four times higher prevalence of OSA in males than in females (*p* < 0.001), but both sexes exhibited the same severity of the disease [11]. Tufik et al. reported 4.1-fold higher odds of developing OSA in men than in females (*p* < 0.001) [12].

#### 2.1.2. Advanced Age

Age is also an important predisposing factor for OSA. Tufik et al. found that the odds ratio of having OSA is 3.9 (*p* < 0.01) for 30–39-year-old people, 6.6 (*p* < 0.01) for 40–49-year-old patients, 10.8 (*p* < 0.01) for 50–59-year-old people and finally 34.5 (*p* < 0.01) for 60–80-year-old people as compared to a 20–29-year-old person [12]. This shows a clear relationship between age and OSA. This data fits well with the results from Eikermann et al. who showed that increased age was linked to both an elevation in pharyngeal collapsibility (*p* < 0.01) and an increase in pharyngeal resistance during sleep (*p* < 0.01) [13].

#### 2.1.3. Obesity and High Body Mass Index 

Heinzer et al. reported a 1.82-fold higher risk of getting mild to severe sleep-disordered breathing (SDB) if the person is a man with a BMI between 25–30 kg/m^2^ compared to a man with a BMI < 25 kg/m^2^ (*p* = 0.0132). They also found a 4.18-fold higher risk of getting mild to severe SDB if the person is a man with a BMI > 30 kg/m^2^ compared to a man with a BMI < 25 kg/m^2^ (*p* = 0.0062). In addition, a woman with a BMI between 25–30 kg/m^2^ has a 3.25-fold higher risk of getting mild to severe SDB compared to a woman with a BMI < 25 kg/m^2^ (*p* < 0.0001). A woman with a BMI > 30 kg/m^2^ has a 2.43-fold higher risk for mild to severe SDB (*p* < 0.011) compared to a woman with a BMI < 25 kg/m^2^ [14]. Furthermore, a weight change has an enormous effect on the AHI and the odds of getting SDB [15].

#### 2.1.4. Other Predisposing Factors

Moreover, menopause in women, various abnormalities of structures of the head and neck, an exaggerated ventilatory response to a respiratory disturbance, endocrine disorders like hypothyroidism, Down syndrome and some neurological disorders are predisposing and precipitating factors of OSA [7].

## 3. Hypertension

There are some differences between the American College of Cardiology (ACC) and American Heart Association (AHA) guidelines for HT and those from the ESH, and this review will follow the locally applied recommendations of the ESH [4,16]. The definition of HT depends on the age group and possible sickness, and the ESH defines HT in general as an SBP > 140 mmHg and/or DBP > 90 mmHg. Patients with HT above these values can benefit from antihypertensive medication (AHM). It is important to lower blood pressure (BP) because it increases the risk of cardiovascular disease (CVD) [4]. Cardiac output and total peripheral resistance determine BP, but HT is a multifactorial disease, which is affected by genetics and lifestyle, among others. HT can be divided into essential and secondary HT, where the majority of hypertensive patients have essential HT with no underlying identifiable cause. It has been shown that 5–15% of hypertensive patients have secondary HT, where the cause of the HT is known [4,17]. The ESH divides HT into the different grades, which are listed in Table 2.

### 3.1. How Can Obstructive Sleep Apnea Cause Hypertension?

OSA is a cause of secondary HT, and the risk for HT increases with the severity of OSA. Some researchers have found a dose-response association between SDB at baseline and HT at the end of the follow-up periods of their studies [3,18]. The HT of OSA patients can be treated by the same pharmacological strategies and with the equal effect as HT caused by other reasons than OSA [19].

Since OSA is a complex disease, there are several reasons why BP will rise. Because of hypoxia, sympathetic activity will be increased by the activation of the arterial chemoreceptors, and this will lead to peripheral vasoconstriction, which will increase BP. Furthermore, patients with OSA have a potentiated peripheral chemoreflex response to hypoxia [20]. This sympathetic activation also leads to an activation of the renin-angiotensin-aldosterone system (RAAS), which will increase the levels of aldosterone and plasma angiotensin II, eventually leading to a rise in BP [21].

Patients with OSA also exhibit an endothelial dysfunction. Vasoconstriction and vasorelaxation are unbalanced due to a decreased level of the endothelium-dependent vasodilator nitric oxide (NO). The now decreased antioxidant capacity will lead to a simultaneous rise in oxidative stress, inducing inflammation, which elevates BP [22,23].

**Figure 1:**
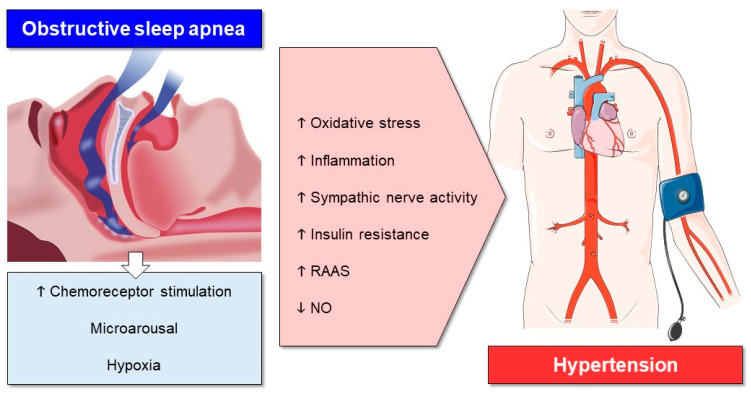
The mechanisms of OSA that lead to hypertension. ↑: elevated; ↓: reduced. Parts of this figure were produced using graphical elements from Servier Medical Art (available from https://smart.servier.com/), licensed under a Creative Commons Attribution 3.0 Unported License.

At the end of the obstructive respiratory event, there will usually be a microarousal. These microarousals will increase sympathetic activity, and together with oxygen desaturation, this will result in a fragmented sleep. Both the increased sympathetic activity and the fragmented sleep will result in an elevated BP [24]. In addition to the reasons already mentioned, OSA induces insulin resistance, which can also cause a rise in BP [25]. The different mechanisms of OSA leading to HT are shown in Figure 1.

BP of OSA patients often follows a nocturnal nondipping pattern [26,27], which has been shown to be associated with cardiovascular events, coronary events, strokes, cardiovascular mortality and mortality (hazard ratios are 1.57 to 1.89 compared to patients with a dipping nocturnal BP) [28].

On the molecular level, it has been found that hypoxia-inducible factor 1α (HIF-1α) serum protein concentration was chronically significantly increased in patients suffering from OSA compared to a healthy control group [29,30]. HIF-1α increases NADPH oxidase 2 (*NOX2*) mRNA expression, resulting in elevated reactive oxygen species (ROS) abundance, one of the risk factors contributing to the development of hypertension [31]. Furthermore, impaired HIF-1α serum levels have been implicated in a circadian clock disruption [32] and as a mediator of insulin resistance, type 2 diabetes [33] and consequently of diabetes-related hypertension [34]. Lastly, it has been shown that HIF-1α is upregulated in pulmonary artery smooth muscle cells of chronically hypoxic mice, driving pulmonary vascular remodeling and pulmonary arterial hypertension [35]. Other studies have proposed an involvement of activated platelets in the development of OSA-related cardiovascular complications [36] and a post hoc miRNA analysis has identified three miRNAs (miR-378a-3p, miR-100-5p and miR-486-5p) that were associated to a favorable CPAP treatment outcome of OSA-related HT. The pathways mostly affected by these miRNAs are the cardiac hypertrophy and NF-kB signaling pathways [37].

### 3.2. The Standard Pharmacological Treatment of Hypertension

The treatment of HT always comprises lifestyle advice, irrespective of the HT grade. The recommended changes in lifestyle are weight loss, regular physical exercise, smoking cessation, moderation of alcohol and dietary changes like sodium restriction and high consumption of fruits and vegetables. Patients with grade 2 or 3 HT will receive pharmacological treatment. High-risk patients with a high normal BP or grade 1 HT suffering of diabetes mellitus and/or CVD will be treated with AHM [4].

AHM is important because a decreased BP is correlated with a lower risk for CVDs including stroke and death [4,38]. The ESH recommends five major drug classes to treat HT: beta-adrenoceptor antagonists (BAA), calcium channel blockers (CCBs), angiotensin-converting-enzyme (ACE) inhibitors, ARBs and diuretics [4].

The ACE inhibitors and ARBs lower BP and the risk of CVD events and mortality. These drugs inhibit the RAAS and can delay the progression of chronic kidney disease and lower albuminuria. The drugs can also prevent small artery remodeling and reduce atrial fibrillations and left ventricular hypertrophy [4,39,40].

CCBs can reduce BP which leads to reduced stroke risk, reduced left ventricular HT, reduced proteinuria and slowed progression of carotid atherosclerosis [4,41]. Diuretics seem more effective than other drug classes to prevent heart failure, and they can reduce BP, which leads to a reduction in CVD events and mortality. The diuretics also lower the serum potassium level but have worse side effects than ACE inhibitors and ARBs [4,42].

BAAs inhibit the beta-adrenoceptors and can significantly reduce the risk of stroke (even though BAA are less effective to prevent stroke than other AHM), heart failure and other CVDs. BAAs have less favorable AEs than RAAS inhibitors but are especially useful in treating HT with symptoms of angina pectoris, tachycardia, heart failure with reduced ejection fraction and infarction, and they can be used as a substitute for ACE inhibitors or ARBs when a woman wants to get pregnant [4,38].

## 4. Continuous Positive Airway Pressure (CPAP)

**Figure 2:**
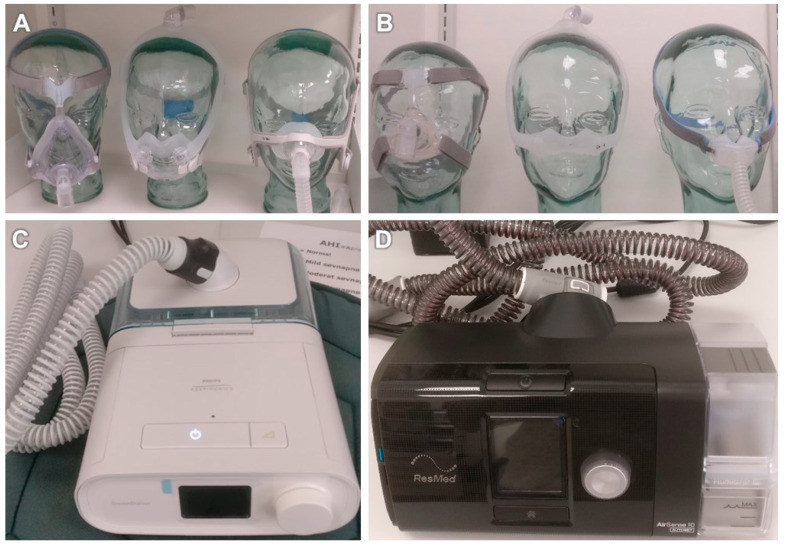
Continuous positive airway pressure (CPAP) machines and masks. (**A**) Nasal and oronasal masks, (**B**) nasal masks, (**C**) Philips CPAP machine and (**D**) ResMed CPAP machine.

According to the guidelines about OSA treatment established by both the AASM and the European Respiratory Society, the gold standard treatment is CPAP [43,44]. Patients diagnosed with moderate-to-severe OSA after a home sleep apnea test or an in-laboratory sleep test should start using CPAP right away in order to improve sleepiness and quality of life and to lower BP [44]. CPAP is a flow generator, which can produce a greater air pressure than the surrounding air. The machine is connected to either a nasal, intranasal or oronasal facemask by a tube, and the excess pressure produced by the machine can generate airway patency, which will counteract the apnea or hypopnea the OSA patient experiences. The most recommended mask is the nasal facemask because patients are most adherent to the CPAP treatment with this mask. It is also recommended that the CPAP is humidified because this reduces several AEs. The CPAP is used by the OSA patient while sleeping and it will reduce the number of obstructive events, oxygen desaturation and sleep fragmentation. The decrease in sleep fragmentation will help the OSA patient to overcome OSA symptoms. In addition to the CPAP treatment, OSA patients are also advised to lose weight, cease smoking and avoid alcohol [44,45]. Examples of CPAP machines and facial masks can be seen in Figure 2.

There are other methods and devices suitable to treat OSA. These include increasing the pharyngeal space with oral appliances if the OSA patient is intolerant to the CPAP therapy. However, oral applications are less effective than CPAP [45,46]. Another treatment option of OSA is surgical modifications of the upper airway to increase the airway surface area and to remove the airway obstruction, but many surgical techniques only partially correct SDB in contrast to CPAP, which exhibits a much higher success rate [45,47]. A further option is medical therapy for OSA, but only topical nasal corticosteroids are recommended by the AASM [48].

### 4.1. The Effect of Continuous Positive Airway Pressure on Hypertension

CPAP treatment showed a decreasing effect on BP in several studies. Kartali et al. found after three months of CPAP therapy a reduction in mean SBP from 141.5 ± 12.1 to 133.5 ± 9.7 mmHg (*p* = 0.007) (only during the night) and a decrease in DBP from 87.8 ± 6.8 to 83 ± 1.4 (*p* = 0.004) (during day and night). Furthermore, heart rate decreased in OSA patients to the frequency of the normotensive control group [49]. Hoyos et al. measured a significantly reduced BP after CPAP therapy as well, which was not influenced by daytime (morning or evening). They also reported a reduced mean central SBP of −4.1 mmHg (*p* = 0.003), mean central DBP of −3.9 mmHg (*p* = 0.0009), mean peripheral SBP of −4.1 mmHg (*p* = 0.004) and a decreased mean peripheral DBP of −3.8 mmHg (*p* = 0.001) [50]. Moreover, Huang et al. examined patients with coronary heart disease and OSA and measured a significantly reduced SBP of 5.6 mmHg (*p* < 0.001) and DBP of 3.0 mmHg (*p* = 0.009) [51].

Yang et al. reported a correlation between CPAP adherence and morning mean BP (MBP) over the four years the study lasted. In patients with high adherence to the CPAP therapy (> 4 h of use per night for > 70% of the nights monitored), there was decreased MBP from 100.8 ± 13.6 mmHg to 96.6 ± 10.8 mmHg (*p* = 0.004). In contrast, patients with no CPAP treatment exhibited a significant increase in MBP [52]. Furthermore, Yang et al. detected a significantly decreased ESS score (from 11.1 ± 5.6 to 9.5 ± 4.9 (*p* = 0.006)), AHI (from 54.8 ± 21.2 episodes per hour of sleep to 39.7 ± 21.4 episodes per hour of sleep (*p* < 0.001)) and oxygen desaturation index (ODI) (from 46.8 ± 24.8 episodes per hour to 33.9 ± 20.5 episodes per hour) in the high CPAP adherence group. Meanwhile, in the no-treatment group there was no significant change in ESS score or AHI, but there was an increased ODI from 35.0 ± 21.4 episodes per hour to 42.7 ± 24.1 (*p* = 0.02) [52].

Huang et al. also found a significantly reduced ESS score after CPAP therapy [51], while Møller et al. showed a significant difference in AHI and ODI between a group of OSA patients without CPAP therapy and a group of OSA patients with CPAP therapy. AHI was 30.1 ± 5.8 episodes per hour of sleep for the group without CPAP therapy and 0.9 ± 0.3 for the group with CPAP therapy (*p* < 0.001), and ODI was 34.9 ± 5.1 for the group without CPAP therapy and 7.4 ± 2.9 for the group with CPAP therapy (*p* < 0.001) [21].

OSA can lead to oxidative stress and a decreased amount of NO. This results in HT [22,23]. CPAP affects both the level of NO and the oxidative stress positively so that BP is decreased. Ip et al. found a significant increase in the level of serum NO after one night of CPAP therapy. The level of serum NO was not significantly different from the level of serum NO of the healthy control subjects [22]. In addition, Barceló et al. reported a significantly elevated total antioxidant status after 12 months of CPAP therapy, while the γ-glutamyl transferase was decreased significantly from 36 ± 15 U/L to 30 ± 14 U/L, and none of these changes differed from those of the healthy control group [23]. Schulz et al. demonstrated in OSA patients after 4.8 ± 0.6 months of CPAP therapy that the concentration of superoxide in neutrophils isolated from blood samples was significantly reduced [53]. The reduction in superoxide concentration and γ-glutamyl transferase as well as the increased total antioxidant status lead to lower oxidative stress and therefore to a lower BP.

### 4.2. Comparison between the Effects and Side Effects of Continuous Positive Airway Pressure and the Standard Pharmacological Treatment of Hypertension

Focusing on the effect of CPAP treatment on HT, Kartali et al. and Hoyos et al. found a reduction in SBP of −8 and −4.1 mmHg and a decrease in DBP of −4.8 and −3.9 mmHg, respectively [49,50]. Several studies reported on the efficacy of AHM in OSA patients. Zou et al. studied the ACE inhibitor enalapril and found a significant reduction in SBP of −12.6 ± 15.9 mmHg and DBP of −8.9 ± 6.5 mmHg in OSA patients with HT [54].

In addition, Pépin et al. have studied the effect of the ARB valsartan in OSA patients with HT who had never used AHM and CPAP. The authors measured a reduction in 24-h SBP of −10.6 mmHg (*p* < 0.001), 24-h DBP of −8.4 mmHg (*p* < 0.001) and a decrease in 24-h MBP of −9.1 ± 7.2 mmHg (*p* < 0.001). Valsartan did also significantly reduce both daytime and night-time SBP, DBP and MBP as well as 24-h heart rate. Moreover, Pépin et al. compared the effect of valsartan with CPAP therapy and detected an overall significant difference in the 24-h, daytime and night-time SBP, DBP and MBP, but not in the 24-h heart rate, where valsartan decreased BP more than CPAP therapy [55].

In summary, these studies show that AHM has the most decreasing effect on BP, but CPAP therapy still has an effect when applied alone.

Almost all treatments have AEs and neither CPAP therapy nor AHM can avoid that. The AEs of CPAP therapy are listed in Table 3, while the AEs of AHM are given in Table 4.

AHM has a significantly better effect on SBP, DBP and MBP than CPAP, but there is evidence that the use of AHM in combination with CPAP has an additional positive effect. Pépin et al., investigating OSA patients with HT who never used AHM or CPAP, showed that 61% of the patients, adherent to CPAP therapy, were not treated sufficiently during all 24 h by monotherapy of ARB or CPAP. Therefore, they applied a combination of ARB and CPAP therapy, and this induced a significant reduction in office SPB, DBP and MBP compared to the ARB and CPAP groups alone [55]. Thunström et al. studied a population of OSA patients with newly diagnosed HT and gave one group losartan (an ARB) and another group both losartan and CPAP. The BP-lowering effect of losartan alone was significantly smaller in patients with OSA compared to those without. Furthermore, they observed a significant reduction in mean 24-h SBP, DBP and MBP in patients treated with losartan + CPAP for >4 h per night (per protocol-population) vs. losartan alone. In the intention-to-treat population, they only found a significant decrease in mean night-time SBP and mean morning SBP [62].

These studies show that the combination of AHM and CPAP has an added positive effect on OSA patients with HT, but this effect might only be seen when the patient is adherent to the treatment.

### 4.3. Clinical Trials Investigating the Effect of Continuous Positive Airway Pressure in Treatment of Hypertension

An overview of the latest clinical trials of OSA and HT is given in Table 5. Most of the studies focus on the effect of CPAP therapy.

## 5. Discussion

The studies mentioned above show that both AHM and CPAP significantly reduce BP. Even though CPAP supports the lowering of BP, AHM is indicated in patients with OSA and hypertension because AHM lowers BP significantly more than CPAP therapy. However, CPAP therapy also has some other beneficial effects like lowering the ESS score, AHI and ODI [52]. Furthermore, it increases the quality of life [2]. Therefore, the use of CPAP therapy is highly relevant in OSA patients.

As already published earlier by Yang et al., the patients’ adherence is important, as CPAP therapy only induces beneficial effects on MBP and sleep quality when the OSA patients were adherent to the treatment [52]. A meta-analysis by Rotenberg et al. showed that between 1994 and 2000, nonadherence rates only slowly improved from about 50% to about 30%, where they remained virtually unchanged until the study ended in 2015, despite many efforts in patient coaching and improvements on CPAP machines and masks [65]. To a certain extent, the adherence can be influenced by AEs, and when comparing the AEs of CPAP therapy and AHM, the AEs of the AHM are more severe than those for CPAP. The AEs of AHM can have a devastating impact on a person’s health and quality of life, while the AE of CPAP therapy are only minor irritations and pains. The severity of AEs is quite important when considering adherence to therapy. Lipophilic BAAs will especially be a problem for OSA patients because their CNS-related AEs can negatively affect sleep. However, due to the multitude of different antihypertensive drugs, it is possible to switch to another medication upon the onset of any given severe AE in order to maintain compliance without compromising the pharmacological therapy as whole.

CPAP therapy is always indicated against OSA irrespective of existing HT because of its beneficial effects on ESS score, AHI and ODI among OSA patients. CPAP therapy might be enough in lowering grades of HT to a normal BP, but it does not seem capable of lowering higher grades sufficiently. Therefore, the combined effect of AHM and CPAP therapy can be used in patients where CPAP therapy is not effective enough to lower BP to a normal range. 

## 6. Methods

For this review, a literature search was performed by using online repositories and clinical trials. The literature search was performed on PubMed (pubmed.ncbi.nlm.nih.gov), scopus.com and clinicaltrials.gov. The search included only English, Danish and Norwegian papers, which can lead to language bias, and because the search only included published papers, there is a risk of publication bias. Additional papers were found in “similar articles”, “cited by” and reference lists of the primary articles.

## 7. Conclusions and Outlook

OSA is an important cause of developing HT. HT can lead to severe CVDs. Therefore, the treatment of both OSA and HT is very important. To a certain degree, HT can be treated with CPAP, but AHM was shown to lower BP significantly more than CPAP therapy. In some studies, CPAP therapy revealed effects on the nightly BP only, while AHM showed an impact on the 24-h BP. The superior effect of AHM is accompanied by more severe AEs than CPAP treatment. Depending on the drug, examples for AEs are renal dysfunction, flushing, edema and hyperkalemia, whereas the severest AEs of CPAP therapy are a bleeding nose, pain in the sinuses, headache and a dry mouth. In parallel, CPAP treatment can lower BP and decrease ESS score, AHI and ODI as well as increase quality of life. These parameters are very important for OSA patients. Therefore, CPAP therapy is still very beneficial for OSA patients.

In conclusion, the best treatment of OSA patients with HT is a combination therapy of both AHM and CPAP therapy, which has demonstrated a significant additive effect on BP, and CPAP has improved the well-being of OSA patients.

OSA will continue to be a major health issue. Because of the risk to develop HT, it is important to diagnose OSA earlier than we do today, since this will reduce the risk of getting CVDs. Furthermore, it is important to study the exact mechanisms leading to OSA and the mechanisms that lower BP while using CPAP. If the mechanisms that lead to OSA are known, OSA can be diagnosed before it induces HT. When the mechanisms for lowering BP while using CPAP treatment are known, then this knowledge can improve HT therapy in OSA patients.

CPAP therapy and AHM have AEs, which can diminish the patients’ adherence to the therapy. Therefore, developing novel AHM with fewer AEs might be favorable because this can heighten the patients’ well-being and quality of life. Moreover, further improvements of CPAP machines are necessary so that more patients will tolerate it.

## Figures and Tables

**Table 1:** The Apnea-Hypopnea Index (AHI).

Obstructive Sleep Apnea (OSA) Severity	AHI Per Hour of Sleep
None/Normal	<5
Mild	5–15
Moderate	>15–30
Severe	>30

**Table 2:** The grades of hypertension.

Category	Systolic Blood Pressure (SBP) (mmHg)		Diastolic Blood Pressure (DBP) (mmHg)
Normal blood pressure (BP)	120–129	and/or	80–84
High normal BP	130–139	and/or	85–89
Grade 1 hypertension	140–159	and/or	90–99
Grade 2 hypertension	160–179	and/or	100–109
Grade 3 hypertension	≥180	and/or	≥110
Isolated systolic hypertension	≥140	and	<90

Adapted from [4].

**Table 3:** Examples of adverse effects (AEs) of CPAP therapy.

CPAP without Humidification	CPAP with Humidification
Bleeding nose	Water condensation in the CPAP system
Dry nose	Water condensation on the face
Nasal congestion and discharge	Water condensation in the nose
Headaches	Water condensation in the mouth
Dry mouth	
Dry and sore throat	
Pain in sinuses	
Reduced smell	
Changed voice	

Modified from [44].

**Table 4:** Examples of AEs of the five major classes of antihypertensive medication (AHM).

ACE Inhibitors	Lipophilic Beta-Adrenoreceptor Antagonists (BAAs)	Hydrophilic BAA	Calcium Channel Blockers (CCBs)	Angiotensin Receptor Blockers (ARBs)	Diuretics
Coughing [56]	Difficulties staying asleep [57]	Dyspepsia [58]	Flushing [58]	No significant differences to placebo [59]	Dizziness [60]
Dizziness [56]	Restless nights [57]	Diarrhea [58]	Edema [58]	Headache [61]	Male gynecomastia [60]
Angioedema [56]	Sexual intercourse problems [57]	Fatigue [58]	Fatigue [58]	Fatigue [61]	Male impotence [60]
Renal dysfunction [56]		Dyspnea [58]	Dyspnea [58]	Dizziness [61]	Hyperkalemia [60]
		Dizziness [58]	Dizziness [58]	Back pain [61]	Orthostatic hypotension [60]
		Headaches [58]	Headaches [58]		Dyspepsia [60]

**Table 5:** An overview of the latest clinical trials of OSA and HT.

Title and Identification Number	Participants	Design	Objective	Status/Conclusions
Untreated Obstructive Sleep Apnea Is Associated With Myocardial Injury Independent of Blood Pressure Control in Hypertension (NCT00843583)	98	Observational Case-Only Cross-sectional Study	Find the prevalence of OSA between resistant hypertension patients. The aim is also to assess the relation be-tween the severity of OSA and BP control.	Completed. In patients with difficult-to-control HT, it was common to find unrecognized severe OSA, and the severity of OSA was associated with myocardial injury.
Effects of OSA Treatment on BP in Patients With Resistant Hyper-tension: A Randomized Trial (NCT00812695)	40	Interventional Randomized Parallel assignment No masking Study Phase III	Investigate CPAP therapy effect on BP control in patients with OSA and refractory hypertension. The aim is also to study how CPAP therapy affects arterial stiffness and cardiac remodeling.	Completed. CPAP therapy on patients with resistant HT significantly reduces the daytime BP.
Effects of Continuous Positive Airway Pressure Treatment on Aldosterone Excretion in Patients With Obstructive Sleep Apnoea and Resistant Hypertension: A Randomized Controlled Trial (NCT01508754)	125	Interventional Randomized-Parallel Assignment Single Masked Study Phase IV	Investigate CPAP therapy effect on BP (both ambulatory and clinical) on patients with OSA and resistant hypertension.	Completed. Significantly reduced aldosterone excretion in patients with uncontrolled resistant HT only under optimal CPAP (per protocol group). The effect was borderline significant in the intention-to-treat group [63,64].
Effects of CPAP on “Vascular” Risk Factors in Patients With Obstructive Sleep Apnea and Arterial Hypertension (NCT00801671)	44	Interventional Randomized Crossover Assignment Double-blind Study Phase III	Investigate CPAP therapy effectiveness in treating systemic hypertension.	Completed. In a group of OSA patients with HT, three weeks of effective CPAP therapy gave a significant decrease in office BP, central BP and augmentation index, and there was an improvement of arterial stiffness parameters.
Long-term Effects of Continuous Positive Airway Pressure on Blood Pressure and Prognosis in Hypertensive Patients With Coronary Heart Disease and Obstructive Sleep Apnea: A Randomized Controlled Trial (NCT02059993)	83	Interventional Randomized Parallel Assignment Single Masked Study	Evaluate the effect of CPAP therapy on BP, cerebrovascular events, metabolic disorder and cardiovascular events in patients who were suffering from OSA and coronary heart disease who used conventional treatment.	Completed. In patients with uncontrolled HT, coronary heart disease and OSA, long-term CPAP therapy reduces daytime SBP.

## Abbreviations

AASM	American Academy of Sleep Medicine
ACE	Angiotensin-converting-enzyme
AE	Adverse effect
AHI	The Apnea-Hypopnea Index
AHM	Antihypertensive medication
ARB	Angiotensin II receptor blocker
BAA	Beta-adrenoceptor antagonists
BMI	Body mass index
BP	Blood pressure
CCB	Calcium channel blocker
CPAP	Continuous positive airway pressure
CVD	Cardiovascular disease
DBP	Diastolic blood pressure
ESH	European Society of Hypertension
ESS	Epworth Sleepiness Scale
HIF	Hypoxia-inducible factor
HT	Hypertension
MBP	Mean blood pressure
NO	Nitric oxide
NOX	NADPH oxidase
ODI	Oxygen desaturation index
OSA	Obstructive sleep apnea
RAAS	Renin-angiotensin-aldosterone system
ROS	Reactive oxygen species
SBP	Systolic blood pressure
SDB	Sleep-disordered breathing
